# Acute Intestinal Obstruction

**Published:** 1910-09-10

**Authors:** 


### SPECIAL ARTICLE.

### ACUTE INTESTINAL OBSTRUCTION.

Acute Intestinal Obstruction is a condition which may be brought about by a great variety of causes. It may be produced by the presence of foreign bodies blocking up the lumen of some part of the gut, by pathological conditions of the wall of the gut itself, such as the various forms of stricture of the bowel, or an intussusception, and by conditions involving the gut externally, for instance, bands, and herniæ.

A Table of Causal Conditions.

These various causes of Acute Intestinal Obstruction may be classified under the following headings:

1. *Conditions inside the gut.*—(*a*) Fæcal impaction; (*b*) foreign bodies.

2. *Pathological conditions involving the gut itself.*—(*a*) Simple or malignant strictures becoming acutely obstructed; (*b*) intussusception; (*c*) volvulus.

3. *Pathological conditions external to but involving the gut.*—(*a*) Strangulation by bands; (*b*) strangulation through orifices and internal herniæ; (*c*) kinking of the gut; (*d*) tumours pressing on the gut.

*Foreign bodies* may be defined as substances which are not acted on by the digestive fluids of the alimentary tract. They may be composed of mineral or vegetable substances, and as examples may be mentioned gall-stones; masses of phosphates, *e.g.* of calcium, magnesium, etc.; magnesium salts which have been taken in excess as medicines; masses of vegetable fibre, *e.g.* the indigestible part of oats, cinnamon bark, cocoa-nut fibre, etc. Of these, gall-stones are by far the commonest. They usually block some part of the small intestine, most frequently in the ileo-cæcal region. They must, therefore, be of considerable size. They never get into the intestine through the bile duct—for if they are large enough to obstruct the bowel, they are much too large to take that course—but always ulcerate through from the gall-bladder into the duodenum, and may leave a cholecysto-duodenal fistula. Before this takes place there is usually some local peritonitis which binds the gall bladder to the duodenum so that a general peritonitis is avoided when the walls of the gall bladder and duodenum become ulcerated through.

Gall-Stone Obstruction.

In a typical case in which a gall-stone ulcerated through the gall bladder into the duodenum and caused acute intestinal obstruction and death, it measured $1\frac{3}{4}$ inches in length and a little more than one inch in its transverse diameter. The patient was a very stout woman aged 59, who, three months before her fatal illness, suffered from pyrexia and pain in the side. Vomiting came on six days before death, but there was neither abdominal pain nor distension. At the necropsy the gall-stone was found about thirty inches below the pylorus firmly impacted in the jejunum. Obstruction by gall-stones occurs much more frequently in women than in men, and in nearly 85 per cent. of the cases the age of the patient is over 50. In some a previous history of gall-stones, jaundice, or biliary colic may be obtained, and the patients are, as a rule, stout. Vomiting and abdominal pain occur very early after the onset, and they are usually severe. Fæcal vomiting is frequent and occurs early. Jaundice is rarely present, and it is most unusual for a tumour to be felt.

Gall-stones are really the only foreign bodies which are likely to give rise to acute obstruction; fæcal accumulation, collections of magnesia, phosphate, etc., cause constipation, but rarely death from acute obstruction.

### A Typical Case.

The following is an interesting case, however, of a much more acute nature, and it can be considered as a case of acute obstruction. The patient was a boy aged 11, who was seen on December 15. His bowels had been opened without any difficulty on December 10. On the following evening he attempted to pass a motion but found he could not do so. On the 12th, 14th, and 15th he made several further attempts, but without success, and each try was accompanied by a good deal of pain in the rectum. On making inquiries, it was found that on December 9 he went for a walk near the docks and was present when a cart laden with cinnamon bark was upset. He filled his pockets with bark and on the way home ate a considerable amount of it. On admission, he was suffering from great pain in the lower part of the abdomen and rectum. His pulse was 90 and his temperature was 98.4° F. His tongue was furred and his breath was very foul. A rectal examination revealed the presence of a large hard mass which felt like an enterolith. Purgatives and enemata were of no avail; in fact, the nozzle of a Higginson's syringe could not be introduced for more than half an inch. An attempt was made to clear out the rectum, but it gave so much pain that an anæsthetic had to be administered, and then with a good deal of difficulty and with very free bleeding a hard solid mortar-like mass which nearly filled a porringer of two pints capacity was cleared out by means of a spoon. The mass removed was found to consist almost entirely of small chips of cinnamon bark.

### Stricture Cases.

The next group of causes comprises pathological conditions that involve the gut itself, the first subdivision being simple or malignant strictures becoming acutely obstructed.

A stricture of the intestine usually causes chronic obstruction, but occasionally acute obstruction supervenes, a condition which, when it does occur, is nearly always brought about by some hard substance inside the gut blocking up its lumen at the seat of the stricture. These cases differ from those of obstruction by foreign bodies on account of the fact that if it had not been for the presence of a stricture there would have been no obstruction, the foreign body, the actual cause of the obstruction, being much too small to block the lumen of a healthy piece of gut. A stricture of the bowel may be defined as a narrowing of the lumen of the intestine, the result of morbid changes in its wall. Stricture may be the result of cicatrisation after ulceration, *e.g.* tuberculous, dysenteric, syphilitic, and catarrhal ulceration; after the injuries which may follow strangulated hernia; and also after primary injury to the gut. It does not occur after typhoid fever.

### An Illustrative Case.

The commonest cause of stricture, however, is carcinoma, and the following case well illustrates this form of acute intestinal obstruction.

A woman aged 61 was admitted to hospital for complete constipation. She had been suffering for a year from difficulty with her bowels, and during the last few months had had six attacks of acute abdominal pain, vomiting, and constipation. For the nine days previous to the date of her admission she had suffered from complete obstruction with vomiting and great distension. Soon after admission to the hospital, laparotomy was performed. The colon was found to be very distended, and an artificial anus was made, the transverse colon being opened and attached to the abdominal parietes. The patient gradually sank, however, and died on the following day. At the post-mortem examination a tight carcinomatous stricture of the descending colon, 1½ inches above the sigmoid flexure, was found, with a lumen which would only just admit a No. 12 catheter; the upper part of this stricture was completely blocked by a cherry-stone. In another case a stricture of the sigmoid colon, when it was laid open, showed a deep and narrow transverse ulcer with raised warty edges infiltrated with a growth of cylindrical-celled carcinoma. The patient was a man aged 44, admitted for intestinal obstruction of two weeks' duration, with occasional vomiting. The man had regarded himself as quite well until intestinal obstruction arose, and yet the carcinomatous stricture had clearly been there for many months.

### Stricture of the Small Intestine.

Stricture of the small intestine occasionally occurs; but it is extremely rare in comparison with stricture of the colon and rectum. Now and then, however, one meets with extraordinary cases of multiple tuberculous strictures of the small intestines.

The patient in one such case was a woman aged 31, who was admitted on October 5 for diarrhœa, anæmia, and general œdema. One of her relatives had died of phthisis. When she was 12 years of age she suffered from "mesenteric disease." She had suffered from chronic diarrhœa off and on for over 18 months previous to the date of her admission. The day before her admission the bowels were opened ten times. This was followed by a period of constipation, and then again by diarrhœa. When the latter was checked by astringents, vomiting came on and was associated with constipation; when the vomiting and constipation were treated there was a return of the diarrhœa until death occurred on November 22. At the post-mortem examination numerous calcareous nodules and small masses which were obviously healed tuberculous glands were seen in the abdomen. There were extensive old fibrous adhesions. The intestines were of small size throughout, and a number of strictures were found to have resulted from chronic tuberculous ulcers, the lumen of the gut at the seat of some of the strictures being so small that it would admit the little finger with difficulty.

These cases illustrate the fact that the stricture itself does not give rise to the symptoms of acute intestinal obstruction. In the first case, the acute symptoms were obviously due to the blocking of the lumen of the narrowed portion of the gut by the cherry-stone; in the last case, as long as the motions were fluid, there was no sign of obstruction, but as soon as the diarrhœa was checked signs of obstruction, namely, vomiting and constipation, supervened.

The diagnosis of such cases is not difficult, for usually there is some previous evidence of chronic obstruction, and when in such a case the symptoms become acute, it is nearly always due to the stenosed portion of the gut becoming blocked. Occasionally malignant strictures may give rise to intussusception, and acute symptoms may arise in this way also.

### Intussusception.

Intussusception.—that is to say the invagination of one piece of intestine into the portion immediately adjacent to it—is the commonest cause of acute intestinal obstruction in infants under a year old. Nearly 60 per cent. of intussusception cases occur in patients under 10 years of age, and of these 25 per cent. during the first year, especially at or about the ninth month. Another point of interest is, that boys are much more liable to this form of intestinal obstruction than girls. The chief, if not the essential factor in the causation of the disease is irregular peristalsis, and more particularly if the irregularity is sudden, severe, and local.

Irregular and violent peristalsis may be brought on by diarrhœa or by chronic constipation. It may follow sudden movements of the body. Two instances have been recorded in which it suddenly came on in a child who was being jumped in its father's arms. It may also follow violent muscular exertion and injuries to the abdomen. The presence of undigested masses of food, the local irritation caused by intestinal worms, mucous polypi and other benign tumours, malignant growths, and even an invaginated diverticulum, may be sufficient to bring about intussusception. Polypi attached to the mucous membrane, or tumours involving it, may be caught by the contraction of the gut immediately above it, the part of the gut to which they are connected being dragged down into the portion of gut below it. Treves writes, however, that in 50 per cent. of the cases no obvious cause can be found. He states that polypi are responsible for less than 5 per cent. of the cases.

### Its Varieties.

Intussusceptions may be (1) ileo-cæcal; (2) ileo-colic; (3) enteric; (4) colic; (5) rectal. Of these the commonest variety by far is that which used to be termed ileo-cæcal, but which really starts just about half an inch to an inch above the ileo-cæcal valve, so that it is really ileo-colic. It is just this half inch above the ileo-cæcal valve that makes all the difference in the treatment; inflation suffices to reduce an intussusception as far as the ileo-cæcal value, but laparotomy is required to ensure the reduction of the half inch above it. An intussusception is usually curved in shape on account of the mesentery which is atached to the invaginated portion. As the intussusception increases in size through the entering and middle layers advancing at the expense of the outer layer, the mesenteric vessels become compressed and finally strangulated, a condition of affairs which leads to congestion and œdema of the invaginated portion, and to actual hæmorrhage from the mucous membrane into the lumen of the gut below. Peritonitis may be set up and lead to adhesions between the entering and receiving layers, which may hinder any further increase in the size of the intussusception, may prevent the possibility of its reduction, and as a result of the strangulation of the vessels the invaginated portion has in very rare cases sloughed off and been passed per rectum.

There exists a specimen of a cæcum and the whole of the ascending colon passed per rectum during life. The patient was a boy aged six, who was seized with abdominal pain and vomiting, and eleven days afterwards passed the piece of gut in question. Left entirely to himself he made a complete recovery, and was known to be alive forty years afterwards; needless to say, such a case is a pathological curiosity.

The diagnosis of acute intussusception is not as a rule difficult. The chief indications are a sudden onset, vomiting, spasmodic abdominal pain, and tenderness, an elongated tumour in the course of the colon, palpable in nearly 70 per cent. of the cases, and the passage of the blood and mucus without fæces per anum. A digital examination of the rectum should always be made, for in about 25 per cent. of the cases a tumour may be felt. The children affected are generally the robust and fat ones, not the ill-nourished and marasmic.

### Volvulus.

Twisting of the gut may be produced and cause obstruction in two distinct ways. In the commoner form there is a twisting of the gut about its longitudinal or mesocolic or mesenteric axis, so that at the point where the ends of the coil cross each other the lumen of the gut is obstructed, strangulation follows, and the affected portion of intestine becomes enormously distended. In the rarer form, two loops of intestine are involved, and are either doubled around each other or knotted together so as to produce a similar result. The parts of the intestine which are most frequently affected are the loosely attached portions of the large intestine, especially the sigmoid flexure and the cæcum; the small intestine is, however, occasionally affected in a similar way. Volvulus of the sigmoid flexure is by far the commonest form; nearly 80 per cent. of the cases are of this variety. The following are the most important factors necessary for the production of this condition:—

A long narrow meso-colon and a long loose loop of colon, the ends of which loop are close together. This abnormal state of the sigmoid flexure is most probably the result of chronic constipation, which leads to constant overloading of this part of the large intestine, and may cause it to hang down into the pelvis, drag on the meso-colon, lengthen it by constantly putting it on the stretch, and in consequence may also approximate the two ends of the loop. The acutal twisting may be brought about by some sudden or violent muscular movement, or by some unexpected change in position. In some of the cases the free end of the loop is found to be bound down by old peritoneal adhesions to some distant part of the peritoneal cavity, a condition which may also have helped to cause stretching and elongation of the meso-colon. The view that these abnormal conditions of the gut and meso-colon are congenital is disproved by the appearance of the intestine in young people, and also by the fact that volvulus is of rare occurrence before the age of thirty. One important alteration in the condition of the strangulated loop is its great distension; in addition to this there are usually extensive inflammatory changes, and the loop is full of fluid rather than air. The twist may consist of one, two, or even three turns about its longitudinal axis.

### Other Forms.

In the other form, when two loops of intestine are involved, such, for example, as the sigmoid colon and the small intestine, both loops are unusually long, and freely movable with long mesenteric pedicles. The method of strangulation is best described in the words of Treves:—

"The loop of small intestine falls in front of or across the pedicle of the sigmoid flexure: the flexure then winds itself around the axis formed by the lower coil, it passes upwards in front of the loop of small intestine, and then moves backwards and downwards, so that the free end passes behind the pedicles of the two coils. In this way the abnormal sigmoid flexure forms a complete turn around the coil of lesser intestine. Both segments of the bowel become strangulated, but the occlusion will be most severe in the axial loop."

In volvulus of the cæcum some congenital defect is almost invariably found; the ascending colon may have an abnormally long meso-colon, and the twist may occur about the meso-colic axis, or the cæcum may be bent or twisted about itself. In the small intestine, a loop may have an abnormally long mesentery, and may be twisted in its vertical mesenteric axis, or two loops of small intestine with abnormally long mesenteric attachments may be twisted or knotted about each other.

### A Typical Case.

The following is an interesting case of volvulus of the ileum. The patient was a young man aged twenty-one, admitted on September 18 for pain in the abdomen. On the 17th he was quite well until about an hour after his supper, at which meal he finished up with a large portion of damson pudding. At 10 p.m. he was seized with severe pains in the hypogastric region, which were so bad that just after midnight he came to the hospital. On admission his pulse rate was 80, his temperature 99° F., and his respiration rate 24. He was in great pain, which was paroxysmal in character. The lower part of his abdomen was rigid and tender, but he was not considered to be dangerously ill. At 8 p.m. on the 18th he passed a large mass of undigested damson skins, and with it about a pint or more of blood. After this he became very collapsed. By 9.15 p.m. he was blanched, restless, and extremely ill. Blood in small quantities continued to be passed per anum, but nothing abnormal could be felt per rectum. He gradually became worse, and died at 5.30 a.m. on September 20. The post-mortem examination revealed a volvulus of the ileum; 100 centimetres of the ileum was at the time of the necropsy of a dull red colour, its peritoneum was covered with flakes of lymph, and some of the coils had almost a gangrenous appearance. The affected portion of the gut had a large mesentery which had become twisted one and a half complete turns in its longitudinal axis.

Such profuse hæmorrhage is unusual under such conditions. With reference to hæmorrhage occurring in intestinal obstruction, Treves writes:—"I have met with two or three recorded instances where blood is said to have been passed in acute cases other than intussusception." No further mention of hæmorrhage is made. Its occurrence in volvulus is a rarity.

The diagnosis of volvulus is difficult, and it is seldom made until the abdomen has been opened. The symptoms develop rapidly, vomiting occurs very early, is severe, and soon becomes stercoraceous, and there is rapid development of enormous distension.

(*To be continued.*)

